# Managing rosacea using asynchronous consumer to physician teledermatology as compared to in-person visits: A retrospective study

**DOI:** 10.1016/j.jdin.2024.05.008

**Published:** 2024-06-22

**Authors:** Vrusha K. Shah, Ryan C. Camacho, Joseph C. English

**Affiliations:** Department of Dermatology, University of Pittsburgh Medical Center, Pittsburgh, Pennsylvania

**Keywords:** compare, diagnosis, in-person, management, retrospective, rosacea, teledermatology, telemedicine

*To the Editor:* Rosacea, a common, inflammatory skin condition can present with pustules, flushing, telangiectasias, and erythema with negative impact on health-related quality of life.^1^^,^^2^ Previously, we showed that teledermatology can adequately treat skin conditions including periorificial dermatitis and acne.^3^^,^^4^ To our knowledge, no studies on managing rosacea patients using teledermatology have been reported. Therefore, our retrospective analysis compares diagnostic, treatment, and follow-up outcomes between patients seen via a distinct teledermatology modality, asynchronous consumer to physician teledermatology (TD), and with an in-person dermatologist. TD allows patients to securely send skin images and answers to survey questions to a teledermatologist through a dedicated patient portal. The teledermatologist sends back diagnosis, treatment/management, and follow-up recommendations using the same portal. As this service is asynchronous, teledermatologists recommended an in-person appointment for patients unable to be treated via TD due to poor image quality or patient information.^5^ We extracted data from 220 TD and 80 in-person rosacea cases as recorded in the UPMC Health System Epic electronic medical record from January 2020 to December 2022. Patient demographic factors are shown in Table I. *P* values for continuous and categorical variables were generated using independent sample t-tests and chi-square tests respectively. Among both TD and in-person cohorts, patients were more commonly female (72.7% vs 68.8%; *P* = .499), White (97.7% vs 92.5%; *P* = .074), and insured (88.6% vs 95.0%; *P* = .099). TD vs in-person patients were more likely to be younger (41.12 vs 51.78; *P* < .001), present with new-onset rosacea (65.5% vs 50.0%; *P* = .015), and have a concurrent mood disorder (16.5% vs 8.9%; *P* = .012). Moreover, TD vs in-person patients were less likely to be Asian (0% vs 3.8%; *P* = .018) and have another concurrent dermatologic condition (18.7% vs 27.7%; *P* = .012). Both cohorts similarly reported triggers such as alcohol (8.7% vs 16.7%; *P* = .182), diet (15.6% vs 16.7%; *P* = .795), heat (14.7% vs 10.0%; *P* = .780), makeup/skin products (12.1% vs 6.7%; *P* = .547), stress (10.4% vs 20.0%; *P* = .130), and sunlight (11.3% vs 10.0%; *P* = 1.000), as well as comorbidities including cardiovascular disease (16.5% vs 17.8%; *P* = .702) and obesity (31.9% vs 27.2%; *P* = .249).

**Table I:** Demographic/disease factors for patients with rosacea among teledermatology and in-person cohorts

Variable	TD (*n* = 220)	In-person dermatology (*n* = 80)	*P* value
Type of rosacea			
Erythematotelangiectatic	103, 46.8%	50, 62.5%	**.016**
Ocular	2, 0.9%	0, 0%	1.000∗
Papulopustular	112, 50.9%	30, 37.5%	**.040**
Phymatous	3, 1.4%	0, 0%	.567∗
Sex			
Female (%)	160, 72.7%	55, 68.8%	
Male (%)	60, 27.3%	25, 31.3%	.499
Age at diagnosis			
Mean ± standard error (SE)	41.12 ± 0.849	51.78 ± 1.720	**<.001**
Race			
White	215, 97.7%	74, 92.5%	.074∗
Black	2, 0.9%	0, 0%	1.000∗
Unknown or N/A	3, 1.4%	3, 3.8%	.195
Asian	0, 0%	3, 3.8	**.018**∗
New condition vs follow-up			
New condition	144, 65.5%	40, 50.0%	
Follow-up	76, 34.5%	40, 50.0%	**.015**
Patient-reported triggers			
Alcohol	20, 8.7%	5, 16.7%	.182∗
Caffeine	1, 0.4%	0, 0%	1.000∗
Chocolate	2, 0.9%	0, 0%	1.000∗
Diet	36, 15.6%	5, 16.7%	.795∗
Exercise	15, 6.5%	1, 3.3%	.703∗
Genetic predisposition	2, 0.9%	1, 3.3%	.308∗
Heat	34, 14.7%	3, 10.0%	.780∗
Makeup/skin products	28, 12.1%	2, 6.7%	.547∗
Medications	3, 1.3%	1, 3.3%	.388∗
Stress	24, 10.4%	6, 20.0%	.130∗
Sunlight	26, 11.3%	3, 10.0%	1.000∗
Temperature	24, 10.4%	2, 6.7%	.749∗
Menstrual cycle	7, 3.0%	0, 0%	1.000∗
Wearing mask	9, 3.9%	1, 3.3%	1.000∗
Comorbidities			
Cardiovascular disease	68, 16.5%	34, 17.8%	.702
Diabetes	17, 4.1%	3, 1.6%	.102
Overweight/obese	131, 31.9%	52, 27.2%	.249
Migraine	29, 7.1%	10, 5.2%	.398
Mood disorder	68, 16.5%	17, 8.9%	**.012**
Other dermatologic condition	77, 18.7%	53, 27.7%	**.012**
Rheumatoid arthritis	3, 0.7%	1, 0.5%	1.000∗
Ulcerative colitis	5, 1.2%	0, 0%	.184∗
Hypothyroidism	6, 1.5%	9, 4.7%	**.024**∗
Vitamin D deficiency	7, 1.7%	12, 6.3%	**.003**

Significant *P* values (*P* < .05) are bolded.

*NOS*, Not otherwise specified.

∗ *P* values generated by Fisher’s exact tests were used for variables with at least one expected cell count less than 5.

Dermatologists seeing patients via TD vs in-person changed/modified treatment frequently (96.4% vs 82.5%; *P* < .001), commonly recommending combination topical (metronidazole, sulfacetamide sodium-sulfur), and oral (doxycycline) treatment (28.8% vs 28.4%; *P* = .935) and less commonly prescribing topical only treatment via TD (28.3% vs 38.5%; *P* = .042) as seen in Table II. TD vs in-person patients less commonly followed-up in the timeline requested (12.7% vs 40.0%; *P* < .001) and preferred to follow-up again via TD (28.6% vs 3.1%; *P* = .009). Notably, among those following up with an in-person dermatologist in the timeline requested, diagnosis between initial TD or in-person visit and follow-up was similar (71.4% vs 84.6%; *P* = .416).

**Table II:** Treatment and follow-up statistics among TD and in-person cohorts

	TD (*n* = 220)	In-person dermatology (*n* = 80)	*P*-value
Treatment change/modification recommendation	212, 96.4%	66, 82.5%	**<.001**
Oral treatment	5, 1.4%	4, 3.7%	.222∗
Topical treatment	103, 28.3%	42, 38.5%	**.042**
Combination topical/oral treatment	105, 28.8%	31, 28.4%	.935
Hygiene recommendations	48, 13.2%	5, 4.6%	**.013**
Avoiding a specific topical treatment	19, 5.2%	2, 1.8%	.185∗
Diet modifications	8, 2.2%	3, 2.8%	.721
Avoidance of triggers	76, 20.9%	22, 20.2%	.875
Patient followed-up?			
Yes, in the timeline requested	28, 12.7%	32, 40.0%	**<.001**
Yes, but not in the timeline requested	51, 23.2%	29, 36.3%	**.024**
No	141, 64.1%	19, 23.8%	**<.001**
Average days to follow-up regardless of requested timeline			
Mean ± SE	209.25 ± 22.19	230.33 ± 29.19	.559
Average days to follow-up among those returning in the requested timeline			
Mean ± SE	56.9 ± 7.70	158.25 ± 30.43	**.003**
Follow-up modality among patients following up in the requested timeline			
TD	8, 28.6%	1, 3.1%	**.009**∗
In-person	14, 50.0%	27, 84.4%	**.004**
Telemedicine	6, 21.4%	4, 12.5%	.491∗
Was diagnosis at the first visit and in-person follow-up the same?			
Yes	10, 71.4%	22, 84.6%	.416∗
No	1, 7.1%	0, 0%	.350∗
Diagnosis not addressed	3, 21.4%	4, 15.4%	.679∗

Significant *P* values (*P* < .05) are bolded.

*SE*, Standard error; *TD*, asynchronous consumer to physician teledermatology.

∗ *P* values generated by Fisher’s exact tests were used for variables with at least one expected cell count less than 5.

Our study provides evidence of the use of TD in managing rosacea patients, suggesting similar diagnostic concordance and treatment recommendations to in-person cohorts. Limitations include a predominantly White cohort, retrospective design, and decreased follow-up adherence among TD patients. As our study found a younger population using TD, future studies should focus on encouraging follow-up education among younger patients opting to use teledermatology.

## Conflicts of interest

None disclosed.

## References

[1] Sharma A., Kroumpouzos G., Kassir M. (2022). Rosacea management: a comprehensive review. J Cosmet Dermatol.

[2] Bewley A., Fowler J., Schöfer H., Kerrouche N., Rives V. (2016). Erythema of rosacea impairs health-related quality of life: results of a meta-analysis. Dermatol Ther (Heidelb).

[3] Shah V.K., English J.C. (2023). Store-and-forward outpatient teledermatology improves care for patients with periorificial dermatitis after an initial primary care consultation: a retrospective study. J Am Acad Dermatol.

[4] Onyekweli T., Agarwal A., Jaklitsch E. (2023). Teledermatology isotretinoin management for moderate-to-severe acne reveals similar outcomes to in-person management: a retrospective study. JAAD Int.

[5] Ghias M., Cline A., Safai B., Marmon S., English J.C. (2023). Teledermatology. Updates in clinical dermatology.

